# Development and Validation of Subjective Financial Distress Questionnaire (SFDQ): A Patient Reported Outcome Measure for Assessment of Financial Toxicity Among Radiation Oncology Patients

**DOI:** 10.3389/fonc.2021.819313

**Published:** 2022-02-02

**Authors:** Mukhtar Ahmad Dar, Richa Chauhan, Krishna Murti, Vinita Trivedi, Sameer Dhingra

**Affiliations:** ^1^Department of Pharmacy Practice, National Institute of Pharmaceutical Education and Research (NIPER), Hajipur, India; ^2^Department of Radiotherapy, Mahavir Cancer Sansthan and Research Centre (MCSRC), Phulwarisharif, India

**Keywords:** radiotherapy, financial toxicity, head & neck cancer, reliability, validity

## Abstract

**Background:**

Financial toxicity is a consequence of subjective financial distress experienced by cancer patients as a result of treatment expenditures. Financial toxicity has been associated with poor quality of life, early mortality, and non-adherence. It is evident from the literature that the currently available instruments for the assessment of financial toxicity do not measure coping and support seeking domains. The aim of this study was to develop an instrument for the assessment of financial toxicity among radiation oncology patients that captures and integrates all the relevant domains of subjective financial distress.

**Materials and Methods:**

The study was conducted among Head & Neck cancer (HNC) patients (age ≥18 years) who have completed the radiotherapy either as stand-alone or part of a multimodal treatment. Literature review, expert opinion, and patient interviews were used for scale item generation. The validity and underlying factor structure were evaluated by Exploratory Factor Analysis (EFA) and Confirmatory Factor Analysis (CFA). The reliability and internal consistency of the final scale was assessed using Cronbach’s alpha coefficient.

**Results:**

A total of 17 items were identified for scale development. The preliminary 17-item instrument was administered to 142 HNC patients. Among 142 participants, 85.9% were male and 98.6% were from rural areas. EFA was performed on 17 items and three items were removed (factor loadings <0.5). The remaining 14 items loaded onto three factors (eigenvalue >1) explaining 62.0% of the total variance. The Chi-square goodness of fit test in CFA and the values of other model fit indices, namely, RMSEA = 0.045, SRMR = 0.014, GFI = 0.92, CFI = 0.98, and TLI=0.97 indicate a good model fit suggesting the three-factor model adequately fits the data. The Cronbach’s α for the final 14-item scale was 0.87 indicating excellent reliability and the Cronbach’s α coefficient of all the individual 14 items was ≥0.85 (range 0.85–0.88).

**Conclusion:**

The SFDQ showed excellent validity and reliability. SFDQ captures and integrates all the relevant domains of financial toxicity. However, the provisional SFDQ instrument warrants further larger sample studies for validation and psychometric evaluation in different primary cancer subsites and treatment modalities from multiple cancer centers to improve the generalizability of this instrument.

## Introduction

Financial toxicity is well recognized but is a relatively new concept in oncology and is considered as analogous to physical toxicity to cancer treatment (1). Financial toxicity has been defined as “a potential consequence of subjective financial distress experienced by patients due to cancer related direct and indirect out-of-pocket (OOP) treatment expenditures” (2, 3). Studies conducted in India have reported catastrophic OOP expenditures among 76.5–84% of cancer patients and approximately 40% of the cancer patients in India use borrowings and sell assets as coping mechanisms to pay for cancer related treatment costs (4–6). With low health insurance coverage, OOP spending for purchasing medicines is a major cause of catastrophe and impoverishment at the household level in India (7).

The prevalence of financial toxicity among cancer patients is reported to be 28–48% and 12–62% of the survivors report being in debt as a result of cancer care related expenses (8, 9). Financial toxicity has been associated with negative quality of life (10–12), early mortality (13), non-compliance (14), non-adherence (15, 16), and poor psychological wellbeing (17, 18). Consequently, the assessment of financial toxicity is important to prevent the negative impact on clinical outcomes.

Financial hardship among cancer patients is reported as three broad aspects: material, psychosocial, and behavioral measures which are further divided into six domains of financial toxicity (2, 9). These domains include financial resources, financial spending, psychosocial affect, coping care, coping lifestyle, and support seeking. Three instruments, namely, Financial Index of Toxicity (FIT), Comprehensive Score for financial Toxicity (COST), and Breast Cancer Finances Survey Inventory (BCFS) have been developed for assessment of financial toxicity among cancer patients (19–21). However, these instruments do not represent and measure all the domains of financial toxicity like coping care, coping lifestyle, and support seeking domains. Witte et al. in a systematic review on methods for measuring financial toxicity after cancer diagnosis and treatment recommended using the identified six domains in development of new instruments for measuring financial toxicity (2).

Moreover, the applicability of current instruments in radiation oncology patients is limited as radiation therapy presents with additional aspects and relevant unmet needs more specific to radiation oncology. Radiation therapy is a continuous treatment modality unlike chemotherapy and patients are required to travel to radiotherapy centres for multiple radiation treatments for weeks. Costs associated with travel, lodging and food while undergoing radiotherapy along with lost work/productivity puts extra financial burden on this patient population which has not been addressed by current instruments (22). D’Rummo et al. reported several limitations while using COST measure for assessment of financial toxicity in radiation oncology patients and recommended further research and development of a specific tool for assessment of financial toxicity in radiation oncology setting (22).

The aim of this study was to develop a patient reported outcome measure for assessment of financial toxicity among radiation oncology patients that captures and integrates all the relevant domains of subjective financial distress. In this paper, we report the development of Subjective Financial Distress Questionnaire (SFDQ) and assessment of its validity and reliability.

## Materials and Methods

### Item Generation for Scale Development

The item generation for the preliminary scale development was done in three steps. In the first step, a pool of items was generated related to financial resources, financial spending, psychosocial affect, coping care, coping lifestyle, and support seeking domains of subjective financial distress using literature search conducted *via* PubMed and Google Scholar. In the second step, the items in the pool were reviewed by oncologists and experts for their importance and relevance to the Indian healthcare system. The review panel consisted of two radiation oncologists, two experts from health economics and two academic experts with experience in patient reported outcome measure research. The overlapping, non-applicable and duplicate items were removed by expert opinion to formulate a preliminary scale. In the following step, importance of the items in preliminary scale was assessed by interviewing thirty Head & Neck cancer patients and further items were added to capture the patient perspectives on the financial distress.

### Ethical Consideration and Patient Population

The study was approved by the Institutional Ethics Committee (RMRI/EC/41/2020) and was conducted at the Mahavir Cancer Sansthan and Research Centre (MCSRC), Patna India, from January 1, 2021 to August 31, 2021. Head & Neck cancer (HNC) patients (age ≥18 years) who have completed the radiation therapy either as stand-alone or part of a multimodal treatment were eligible for participation in this study. Patients not able to give informed consent were excluded from this study. Consecutive HNC patients attending radiation therapy out-patient-department on follow-up after radiotherapy completion were approached and patients willing to participate with informed consent were enrolled in this study. All relevant information, namely, socio-demographic and clinical characteristics of a patient were collected from the patient’s record file in a pre-designed case record form. The preliminary17-item instrument was administered by face-to-face interviews.

### Validity and Reliability Analysis

The validity of the scale was evaluated through Exploratory Factor Analysis (EFA) and Confirmatory factor analysis (CFA). EFA evaluates the factor structure according to how participants respond to the scale items. The exploratory factor analysis is essential to determine underlying factor structure for a set of measured variables. Kaiser–Meyer–Olkin (KMO) test and Bartlett’s test of sphericity were performed to determine the sampling adequacy and suitability of the data for performing EFA. To test the underlying factor structure of preliminary instrument, EFA was conducted using principal component analysis (23, 24). The number of underlying factors was identified using Kaiser Criterion (eigenvalues >1), Scree plot and percentage of total variance explained. Eigenvalue is the amount of variance explained by each extracted factor. The items with factor loadings <0.5 were considered significant and retained in the scale (23).

CFA was used to evaluate the underlying factor structure and to determine whether the factors extracted in EFA adequately describe the data. CFA with maximum likelihood method was performed to test the final model fit. Chi-square goodness of fit test (χ^2^), Root Mean Square Error of Approximation (RMSEA), Standardized Root Mean Square Residual (SRMR) and Goodness of Fit Index (GFI) along Comparative Fit Index (CFI), and Tucker–Lewis Index (TLI) were used to evaluate the overall fit of CFA model (23, 24). An acceptable model fit was indicated by a *P-*value of >0.001 in χ^2^ test, RMSEA <0.06, SRMR value of <0.09, a value of ≥0.96 for CFI and TLI, a value of ≥0.9 for GFI (25, 26). Reliability final scale was investigated using Cronbach’s α coefficient for internal consistency and a Cronbach’s α of ˃0.7 was considered acceptable (23). EFA and CFA were performed using SPSS and AMOS version 27.

### Sample Size

The sample size for EFA is very important for reliable factors. According to Osborne and Costello, the most common guideline for suitable sample size in EFA is the ratio of sample size to the number of variables (participant to item ratio) (27). Hair et al. recommend a minimum 5:1 participant to item ratio, and 10:1 as more acceptable sample size (23). A sample size of 150 was considered appropriate for this study, giving the participant to item ratio for this analysis approximately 9:1 (where sample size was 150 and the number of variables included was 17).

## Results

### Item Generation for Scale Development

In order to develop a robust instrument and ensure that all relevant aspects of subjective financial distress are taken into consideration, six domains of subjective financial distress were identified from literature, namely, financial resources, financial spending, psychosocial affect, coping care, coping lifestyle, and support seeking. A thorough literature search was conducted and a pool of 47 items was generated from different scales and self-designed questionnaires measuring one or more identified domains of financial distress/toxicity. These scales include Financial Index of Toxicity (FIT) (19), Comprehensive Score for financial Toxicity (COST) (20), Breast Cancer Finances Survey Inventory (BCFS) (21), Incharge Financial Distress/Financial Wellbeing Scale (28), Socioeconomic Wellbeing Scale (SWBS) (29), Social Difficulties Inventory (SDI) (30), European Organization for Research and Treatment of Cancer Quality of Life Questionnaire (EORTC QLQ-C30) (31), Cancer Care Outcomes Research and Surveillance Consortium (CanCORS) Patient Survey (32), and Short form Patient Satisfaction Questionnaire (PSQ-18) (33).

**Figure 1:**
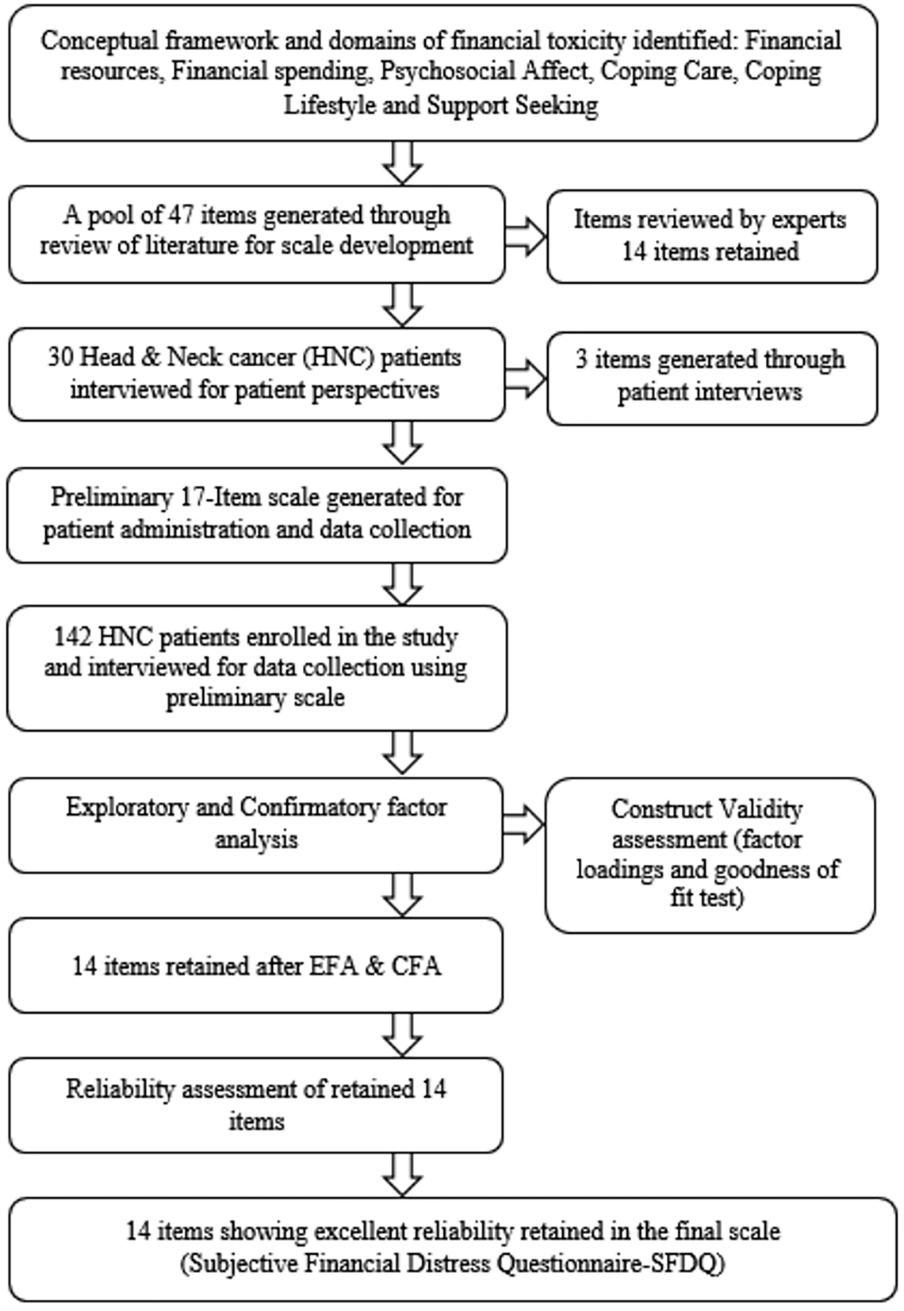
Flowchart of the scale development process.

The 47 item poll was generated with 13 items on financial resources, 7 items on financial spending, 15 items on psychosocial affect, 5 items on coping care, 4 items on coping lifestyle and 3 items on supporting seeking domains (**Supplementary Table 1**). The item pool was screened by review panel (oncologists, health economic and academic research experts) and the items applicable to radiation oncology in Indian health care setting were identified and selected through discussion and mutual consensus. The overlapping (22), non-applicable (3) and duplicate (8) items were removed to formulate a preliminary scale of 14 items. The preliminary scale was further refined by interviewing 30 HNC patients in radiation therapy out-patient department to capture the patient perspectives on financial distress. Based on patient interviews and expert opinion, two items on financial spending and one item on support seeking domains were identified (**Supplementary Table 1**) and added to the preliminary items. The 17 items selected were modified/rephrased and a 17-item preliminary Likert scale was developed for patient administration and data collection. The 17 items were coded as Item-1 to Item-17 (**Supplementary Table 2**). Flowchart in **Figure 1** summarizes the stepwise scale developmental process.

### Sociodemographic and Clinical Characteristics of Participants

Based on inclusion criteria, the 17-item preliminary instrument was administered to 150 HNC cancer patients for data collection using interview method. Eight participants were excluded due to lack of an informed consent. The sociodemographic and clinical characteristics of remaining 142 participants are presented in **Table 1**. Among 142 participants, 85.9% were male and 98.6% were from rural areas. Cancers of oral cavity were the most frequently (65%) diagnosed cancers followed by larynx and oropharynx. Fifty percent of the participants had no education and only 9.2% were employed, 17% were unemployed and more than 40% were working as manual labors and farmers. Majority of the patients (96.5%) had no health insurance and more than 80% applied for government or non-governmental financial aid to cover the cancer related treatment expenses.

**Table 1:** Sociodemographic and Clinical Characteristics (*N* = 142).

Characteristics	N (%)	Characteristics	N (%)
Gender		Current employment Status	
Male	122 (85.9)	Stopped Working	99 (69.7)
Female	20 (14.1)	No Change in Work	26 (18.3)
Age Group		Reduction in Work	17 (12.0)
18–59	97 (68.3)	Primary Cancer Site	
≥60	45 (31.7)	Oral Cavity	92 (64.8)
Residence		Larynx	12 (8.5)
Rural	140 (98.6)	Oropharynx	12 (8.5)
Urban	2 (1.4)	Hypopharynx	9 (6.3)
Marital Status		Salivary Glands	6 (4.2)
Married	135 (95.1)	Paranasal Sinus	3 (2.1)
Unmarried	6 (4.2)	Thyroid	2 (1.4)
Others	1 (0.7)	Others	6 (4.2)
Education Level		Disease Extent	
Not Educated	72 (50.7)	Metastatic	7 (4.9)
Primary level	44 (31.0)	Non-Metastatic	133 (93.7)
Secondary Level	15 (10.6)	Treatment Modality	
Graduate and above	11 (7.8)	RT	17 (12.0)
Occupation		RT + S	59 (41.5)
Employed	13 (9.2)	RT + CT	44 (31.0)
Unemployed	17 (12.0)	RT + CT + S	22 (15.5)
Labour Work	37 (26.1)	Treatment Intent	
Farming	25 (17.6)	Definitive	129 (90.8)
Homemaker	16 (11.3)	Palliative	13 (9.2)
Others	34 (23.9)	Time Since RT	
Annual Household Income (INR)		less than 1 month	26 (18.3)
less than 50,000	56 (39.4)	1–6 months	35 (24.6)
50,000–100,000	51 (35.9)	6–12 months	23 (16.2)
100,000–150,000	17 (12.0)	12–18 months	11 (7.7)
150,000–200,000	6 (4.2)	18–24 months	18 (12.7)
˃200,000	12 (8.5)	˃24 months	29 (20.4)
Health Insurance			
Yes	5 (3.5)		
No	137 (96.5)		

RT, Radiation therapy; CT, Chemotherapy; S, Surgery.

### Validity Assessment

The validity of the scale was assessed by EFA and CFA. Construct validity indicates the extent to which the items in the scale reflect and measure the corresponding domain/factor of financial toxicity. The appropriateness of the data for factor analysis was assessed using Kaiser–Meyer–Olkin (KMO) and Bartlett’s tests (**Table 2**). A KMO of 0.888 and significant Bartlett’s test (*P-*value < .001) indicate the data was suitable to perform EFA. EFA was performed on 17 item preliminary scale using principal component analysis. Factors were extracted based on Kaiser Criterion, Scree plot and percentage of total variance explained. According to Kaiser Criterion, all factors that have an eigenvalue >1 are retained for interpretation. Item factor loadings greater than 0.5 were considered factor specific and factor loadings below 0.5 were suppressed.

**Table 2:** KMO and Bartlett’s Test (*N* = 142).

Kaiser–Meyer–Olkin Measure of sampling adequacy		0.888
Bartlett’s Test of Sphericity	Approx. χ^2^	888.538
	*Df*	91
	Sig. (*p*)	.000**

*p-value significance level ˂0.001.

**Figure 2:**
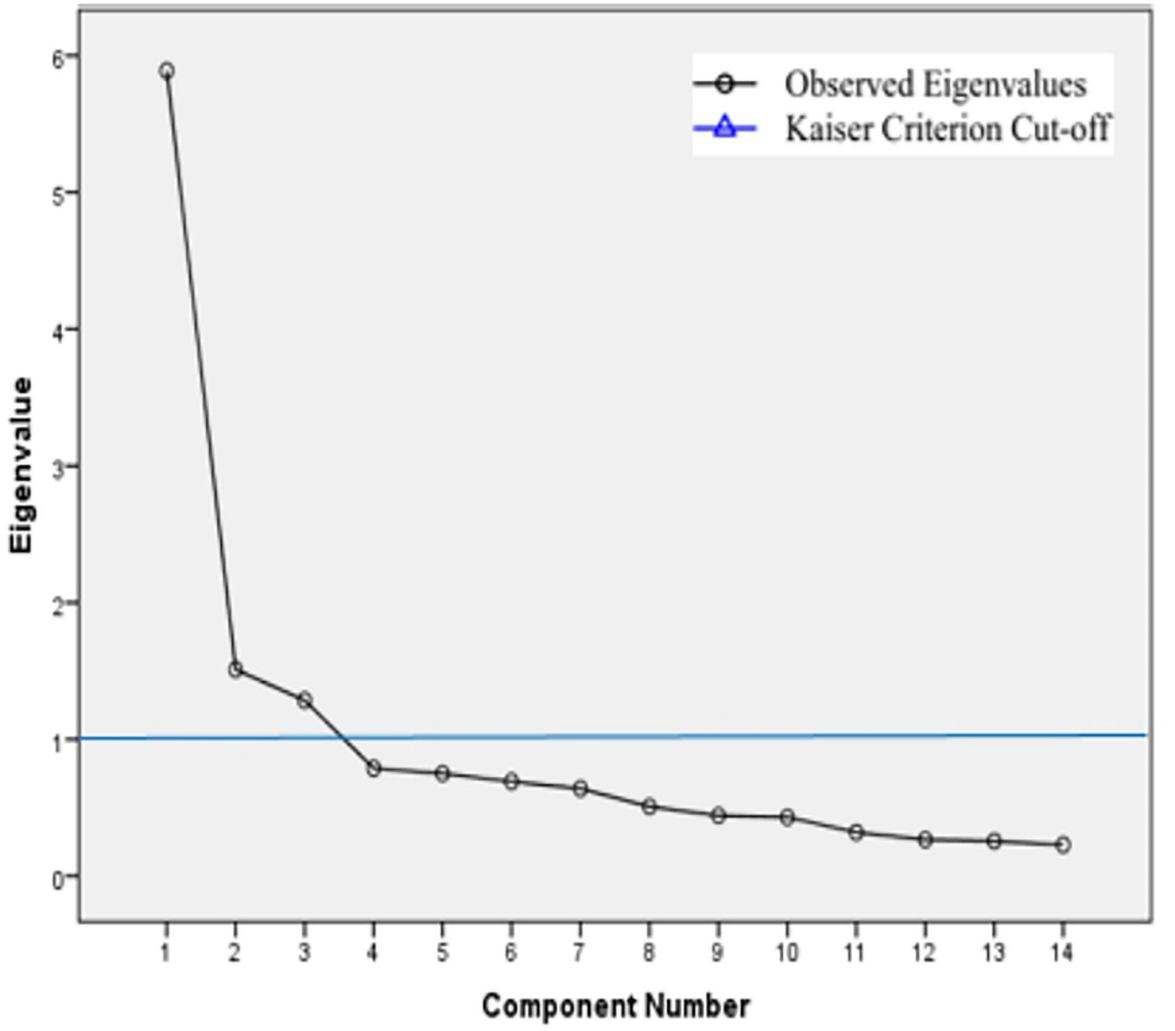
Scree plot with the Kaiser criterion.

Three items (Items 3, 4, and 5) were removed due to low factor loadings and cross-loadings. The remaining 14 items loaded onto three factors explaining 62.0% of the total variance in EFA (**Table 3**). The Scree plot along with the Kaiser criterion for determining the number of significant factors is shown in **Figure 2**. Looking at **Figure 2**, there were three factors with eigenvalue >1. On the basis of percentage of total variance explained, scree plot and Kaiser Criterion, three factors were extracted in EFA. The factor loadings of the retained 14 items are shown in **Table 4**. Based on the results of EFA, 14 items were retained for further analysis.

**Table 3:** Eigenvalues and percentage of total variance explained in exploratory factor analysis.

Component	Initial Eigenvalues			Extraction Sums of Squared Loadings			Rotation Sums of Squared Loadings		
	Total	% of Variance	Cumulative %	Total	% of Variance	Cumulative %	Total	% of Variance	Cumulative %
1	5.889	42.068	42.068	5.889	42.068	42.068	5.626	40.188	40.188
2	1.510	10.787	52.854	1.510	10.787	52.854	1.557	11.122	51.310
3	1.285	9.177	62.032	1.285	9.177	62.032	1.501	10.722	62.032
4	.788	5.626	67.658						
5	.750	5.356	73.014						
6	.691	4.935	77.949						
7	.639	4.568	82.517						
8	.508	3.625	86.142						
9	.442	3.154	89.296						
10	.430	3.073	92.369						
11	.319	2.282	94.651						
12	.266	1.899	96.550						
13	.256	1.825	98.375						
14	.228	1.625	100.000						

Extraction Method: Principal component analysis with varimax rotation.

**Table 4:** Item Factor loadings in rotated component matrix.

Items	Factors		
	F1	F2	F3
Item1	.685		
Item2	.733		
Item6	.746		
Item7	.859		
Item8	.605		
Item9	.794		
Item10	.633		
Item11	.813		
Item12	.753		
Item13	.817		
Item14		.827	
Item15		.765	
Item16			.775
Item17			.836

Extraction Method, Principal Component Analysis;

Factor-1, Financial and Psychosocial affect;

Factor-2, Coping behavior.

Factor-3, Support seeking.

**Figure 3:**
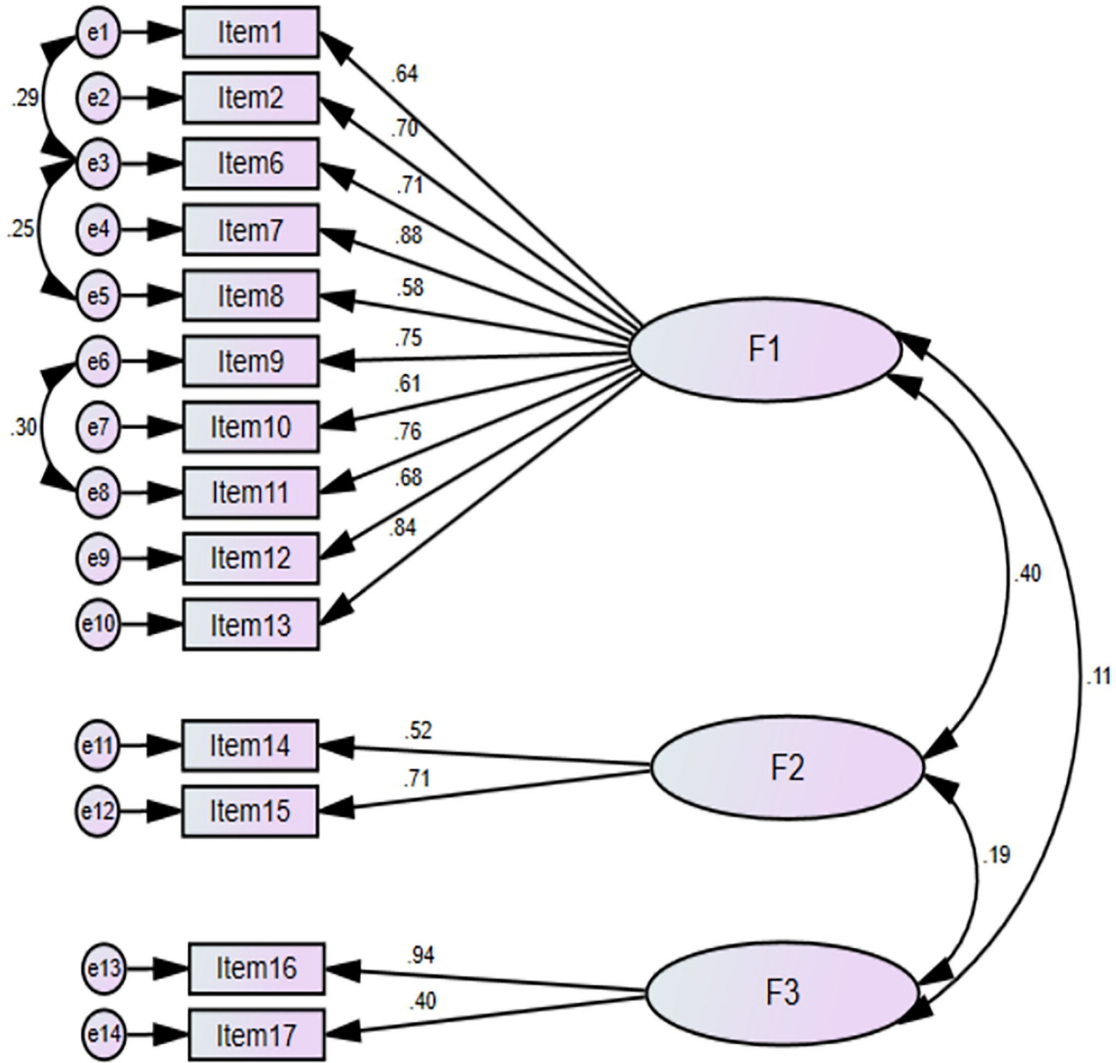
CFA model diagram with standardized regression weights. Factor-1, Financial and Psychosocial affect domain; Factor-2, Coping behaviour domain; Factor-3, Support seeking domain.

CFA with maximum likelihood estimation method was performed for 14 items retained in EFA to assess the underlying factor structure and to determine whether the factors F1, F2, and F3 adequately describe the data. The CFA model diagram is presented in **Figure 3**. The results of CFA showed that χ^2^ (71) = 91.42, *P* = 0.052 indicating χ^2^ test was not significant (*P* >0.001) suggesting that the CFA model adequately fits the data. The value of Satorra–Bentler scaled chi-square statistic to degree of freedom ratio (χ^2^/*df*) = 1.28 also indicates an excellent model fit. The values of other model fit indices including RMSEA = 0.045, SRMR = 0.014, GFI = 0.92, CFI = 0.98, and TLI = 0.97 are all indicative of a good model fit. Based on the results of CFA, 14 items were retained in the scale for reliability analysis. The results of EFA followed by CFA suggest excellent construct validity of 14-item scale.

Discriminant validity indicates the extent to which the extracted factors are distinct from each other. The discriminant validity was evaluated using Fornell–Larcker criterion. According to this technique, discriminant validity is supported if the square root of average variance extracted (AVE) for each factor is greater than the correlation with other latent factors in CFA model. AVE was calculated by ∑ (Standardized Loadings^2^)/Number of Indicators. The √AVE for each factor and correlation between latent factors is shown in **Table 5**. The √AVE for each factor was greater than the correlation between the factors (F1, F2, and F3).

**Table 5:** Square root of AVE and correlations between factors.

	Factor 1	Factor 2	Factor 3
Factor 1	**0.721**		
Factor 2	0.40	**0.622**	
Factor 3	0.109	0.194	**0.720**

√AVE (diagonally in bold) and correlations between latent factors (off-diagonal).

### Reliability Assessment

**Figure 4:**
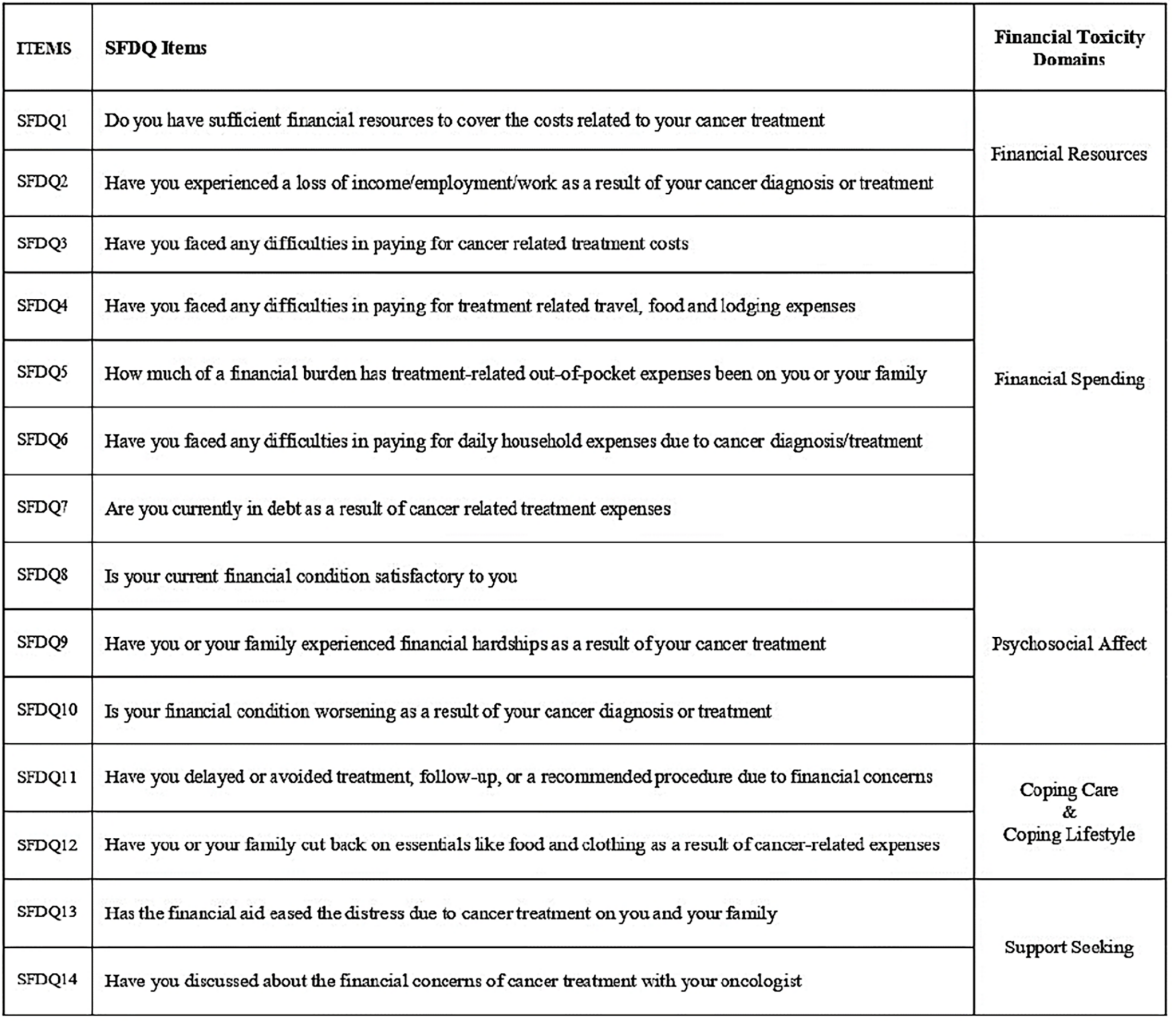
Final 14-item Subjective Financial Distress Questionnaire (SFDQ).

The reliability and internal consistency of the final 14 items retained after EFA and CFA was assessed using the Cronbach’s alpha coefficient. The Cronbach α for the final 14 items was 0.87 indicating excellent reliability. No increase in Cronbach α values was observed if any of the individual items were removed (**Table 6**). The final 14-item Subjective Financial Distress Questionnaire (SFDQ) is shown in **Figure 4**.

**Table 6:** Cronbach’s α, if any individual item was deleted.

Items	Scale Mean if Item Deleted	Scale Variance if Item Deleted	Corrected Item-Total Correlation	Cronbach’s Alpha if Item Deleted
Item1	14.13	21.464	.649	.857
Item2	14.41	20.697	.622	.856
Item6	14.30	20.510	.697	.853
Item7	14.65	18.201	.803	.844
Item8	14.32	21.651	.558	.861
Item9	15.11	20.469	.684	.853
Item10	14.61	20.794	.558	.860
Item11	14.13	21.246	.708	.855
Item12	14.09	21.786	.598	.860
Item13	14.72	19.651	.768	.847
Item14	15.46	22.576	.248	.877
Item15	15.44	22.135	.322	.874
Item16	15.08	23.581	.167	.876
Item17	15.08	24.058	.058	.880

## Discussion

The screening of cancer patients at risk of developing financial toxicity is crucial for better clinical outcomes and policy development to alleviate the financial burden on cancer survivors.

Radiation therapy is an important treatment modality and nearly two-thirds of cancer patients require radiation therapy either as a single or part of a multimodal therapy (34). Financial toxicity among radiation therapy patients presents with additional aspects more specific to radiation oncology (22). Radiation therapy is a one-time continuous treatment unlike chemotherapy and patients are required to pay the entire cost in advance. A typical radiation therapy course lasts for 5–7 weeks depending on treatment intent therefore the patients and caregivers need to stay near the radiation therapy centre for longer periods of time or travel daily until treatment completion. Consequently, patients have to bear transportation, food and lodging costs along with the loss of productivity. Mohanti et al. reported more than 55% of the costs during radiation therapy were related to food, lodging and transportation (34). These specific unmet needs in radiation oncology patients have not been adequately addressed by current instruments.

It is evident from literature that the currently available measures of financial toxicity do not measure all the relevant domains of subjective financial distress (2). To the best of our knowledge, there are no comprehensive instruments available for measuring the subjective financial distress among radiation oncology patients and the applicability of current instruments is limited in this patient population. In this study, we aimed to develop and validate a scale for measuring financial toxicity that captures and integrates all the relevant domains of subjective financial distress among radiation oncology patients.

HNCs account for 30–40% of all the cancers in India particularly cancers of the lip-oral cavity (35). In this study, the cancers of oral cavity, larynx, oropharynx, and hypopharynx were the most frequently diagnosed cancers. Cancers of lip-oral cavity are the second leading cause of cancer incidence and the third leading cause of mortality in India (36). Majority of the study participants were male because cancers of lip-oral cavity are the leading cause of incidence and mortality among men in India (36). Financial toxicity is a major concern in HNC survivors and studies show that 33% of HNC patients stop working and 48% lower their workload after treatment (37). In this study, 69.7% of the patients stopped working entirely and 12% reduced the workload after radiation therapy. Financial toxicity in HNC is disproportionately higher as compared to other cancers due to high median costs of treatment and long term patient needs due to treatment related functional deficits. Massa et al. in a retrospective study of 16,771 patients reported substantially higher OOP expenses and higher financial toxicity among HNC patients as compared to other cancer survivors (38).

Based on extensive literature search, expert opinion and patient participation, a preliminary 17-item scale was developed measuring six domains of financial toxicity including financial resources, financial spending, psychosocial affect, coping care, coping lifestyle, and support seeking. The validity (construct and discriminant validity) was evaluated by EFA and CFA. The overall methodology for the development of this new instrument was in line with the methodology used in development of FIT and COST instruments.

### Factor Extraction and Interpretation

KMO test measuring sampling adequacy and Bartlett’s test measuring the significance of all the correlations within the correlation matrix were performed to determine the appropriateness of the data for EFA. Both KMO and Bartlett’s test supported the factorability of the items (23). In EFA three items were removed and the remaining 14 item loaded onto three factors explaining 62.0% of the total variance. Hair et al. suggest a solution accounting for 60% total variance explained as satisfactory for factor extraction (23). The range of factor loadings for 14 items was 0.60–0.86 with no cross-loadings suggesting a factor structure that is valid and easy to interpret. Hueniken et al. and de Souza et al. have used 0.3 and 0.5 threshold for factor loadings respectively in the development of FIT and COST. However, items with factor loadings <0.3 have been retained in FIT as against the cut-off (<0.3) mentioned in their methodology.

Factor-1 comprising of 10-items was classified as “Financial and Psychosocial affect domain”. Factor-2 comprising of 2-items was classified as “Coping behavior domain”. Factor-3 comprising of 2-items was classified as “Support seeking domain”. De Souza et al. and Hueniken et al. reported one and three factors respectively representing material and affect domains in development studies of COST and FIT (19, 20). Factor-1 in this study was comparable to the COST development study by De Souza et al. who reported only one factor and the items in this factor represented financial resources, financial spending, and psychosocial affect domains similar to this study (20).

### Model Fit Evaluation

Confirmatory factor analysis of 14-items retained in EFA was performed to evaluate the underlying factor structure and to determine whether the three factor model (F1, F2, and F3) adequately describe the data. Both Absolute fit measures (χ^2^ test, RMSEA, SRMR) and incremental fit measures (TLI & CFI) were used to evaluate the overall fit of the CFA model. Absolute fit measures are sensitive to sample size hence incremental fit indices were also used (23).

The Chi-square statistic is the most popular statistic used to measure model fit and it is a standard practice for CFA to include the Chi-square test (23–25) The results of CFA showed that χ^2^ test was not significant (*P* ˃0.001) suggesting that the CFA model adequately fits the data indicating a good model fit. However, the χ^2^ test is sensitive to sample size therefore other absolute fit measures along with incremental fit measures were used to evaluate the overall fit of CFA model (23). The values of other model fit indices are all indicative of a good model fit. Hueniken et al. and de Souza et al. have not performed CFA to test the factor structure in development of FIT and COST instruments.

### Instrument Validity and Reliability

Construct validity indicates the extent to which the scale items reflect and measure the corresponding extracted factors. The results of EFA and CFA support excellent construct validity of 14-item SFDQ for assessment of financial toxicity. Discriminant validity indicates the extent to which a factor correlates with the scale items representing that factor as compared to correlation with other factors. Discriminant validity indicates how distinctly the scale items represent a single factor (23, 39). Lack of discriminant validity is questionable and has serious implications on factor structure. Fornell–Larcker criterion is a widely used technique for assessment of discriminant validity (40). The AVE for each factor was greater than the shared covariance with other latent factors indicating that each latent factor explains better variance of its indicator variables than variance of other latent factors. The results support and the discriminant validity is acceptable for this model.

The reliability/internal consistency of the final 14-item scale was assessed using the Cronbach’s α coefficient. The Cronbach’s α for the final scale was 0.87 indicating excellent reliability and the Cronbach’s α coefficient of all the individual 14 items was ≥0.85 (range 0.85–0.88).

### SFDQ Scoring Guidelines

The responses to 14 SFDQ items include 0 (not at all), 1 (somewhat) and 2 (very much) and provides a total score ranging from 0 to 28. Item-1 and Item-8 are reverse coding items as the lower score indicates higher financial toxicity. The answers to the SFDQ items are recorded during patient administration followed by reversals as indicated above. The individual items are added to obtain a score. This score is multiplied with the number of items in the scale and then divided by number of items answered giving the final SFDQ score. The total score (0–28) has been divided into four categories representing different grades of financial toxicity: 0–7 (Grade-1), 8–14 (Grade-2), 15–21 (Grade-3), and 22–28 (Grade-4).

### Comparison With Other Scales

The instruments like FWBS, SWBS, and SDI are generic in nature and not specific to cancer patients while EORTC QLQ-C30, CanCORS, and PSQ-18 are large questionnaires with a subscale for financial burden assessment (28–33). FIT, COST, and BCFS have been developed for assessment of financial toxicity among cancer patients (19–21). However, these scales do not measure all the relevant domains of financial toxicity. The BCFS was designed exclusively for breast cancer patients; therefore its applicability to other cancer subsites is limited or minimal. Both FIT and COST does not assess all the six domains of subjective financial distress as mentioned in introduction. The COST measure has 8 items on psychosocial affect, 2 items on financial resources, 1 item on financial spending and one summary item. Similarly the FIT has 3 items on financial stress, 4 items on financial strain and 2 items on lost productivity. Both these instruments have no items/variables for measuring coping care, coping lifestyle, and support seeking domains of financial toxicity (2, 19, 20). In comparison SFDQ has 14 items representing the material, psychosocial, and behavioral constructs of financial toxicity. SFDQ has 2 items on financial resources, 5 items for financial spending, 3 items covering the psychosocial affect, 2 items covering the coping behavior, and 2 items measuring support seeking domains.

Coping and support seeking behavior are vital domains of subjective financial distress. Cancer patients who are unable to pay for the cost of cancer care resort to different coping mechanisms like coping healthcare (non-compliance, medication non-adherence or forgoing treatment) and coping life style (spending less on basics like food and clothing, borrowing money). Studies have reported 45% cost related medication non-adherence among cancer patients and 69% of the HNC patients use coping strategies to pay for cancer care (37, 41). Coping healthcare with delaying or forgoing recommended treatment has detrimental implication on clinical outcomes.

Support seeking behavior particularly patient–oncologist communication is very crucial to manage the cost of cancer care. Literature shows that only 19% of patients discuss financial concerns with their oncologist while more than 50% of the cancer patients desire to discuss cancer care costs with oncologists (42, 43). Zafar et al. reported that 57% of the cancer patients experienced lower OOP expenses as a result of patient–oncologist cost communication (43, 44). A proper patient–oncologist cost communication has potential to prevent or at least lower the financial toxicity among cancer patients. SFDQ incorporates coping care, coping life style, and patient–oncologist cost communication factors along with financial and psychosocial domains in assessment of financial toxicity.

The COST and FIT instruments have been developed and validated among cancer patients undergoing chemotherapy (19, 20). Radiation oncology is a resource intensive treatment modality and radiotherapy centers are limited due to costly infrastructure and trained personnel requirements. The standard conformal 6–7-week radiation therapy costs INR 50,000 to 75,000 excluding the costs of medications and dietary needs to manage the associated side effects. In this study 98.6% of the patients belonged to rural areas and 39.4% reported an annual income of less than INR 50,000. Cancer patients from rural areas have to bear extra costs of transportation, food, and lodging while undergoing radiation therapy. SFDQ has been specifically developed in radiation oncology patients and captures these specific needs like expenses related to transportation, food, and lodging.

The COST and FIT instruments have been developed in the USA and Canada respectively (19, 20). Majority of the population in these developed countries is covered through health insurance like the Patient Protection and Affordable Care Act in the USA (45). However, in lower-middle income countries (LMICs) like India, the public spending on health care is low. Less than 2% of GDP is spent on public healthcare in India and health insurance coverage is less than 20%. (46) Subsequently, the healthcare system in India is heavily dependent on OOP payments incurred by patients or caregivers (47). The COST measure has only one family statement which is a summary item only and is not considered towards total scoring (20). Thus financial distress on caregivers/families has not been taken into consideration. Consequently, the application of COST is limited when the patient is not the earning member of the family but dependent on caregivers/family. The female and elderly population were dependent on their families for treatment costs in this study. Although FIT has family oriented items, however its applicability is too limited due to lack of coping and support seeking domains of financial toxicity (19). SFDQ has been developed to assess the financial toxicity from the perspective of an individual and family as well.

### Limitations and Future Developments

This development study of SFDQ is the first step in building a robust scale for measuring financial toxicity among cancer patients. The current study has many limitations which will be addressed in further studies. A major limitation of this study was enrolment of patients from one primary cancer site (HNC) from a single institution only which limits the generalization of the results. Thus further validation and psychometric studies are needed in different primary cancers from multiple centers to improve the generalizability of this scale. Second, the small sample size in this study may not represent the entire HNC patient or other cancer site populations. However, the aim of this study was development of a valid and reliable scale. Thus further larger sample studies including diverse cancer patient population are needed for psychometric validation in association with quality of life, symptom burden and other sociodemographic and clinical characteristics. Lastly, the patient reported outcome measures are designed for patient self-administration although interview based administration is also appropriate. Thus additional language translations are warranted to improve the responsiveness and generalizability of this scale. Since SFDQ has been developed in India, further cross-cultural and linguistic validations are required for adaptation and use in different cultures/countries.

### Conclusion

With the extensive literature search, expert opinion and patient participation, Subjective Financial Distress Questionnaire (SFDQ) for measuring financial toxicity among cancer patients treated with radiotherapy was developed. SFDQ showed excellent construct validity and reliability. SFDQ captures and integrates all the relevant domains of subjective financial distress. SFDQ could be useful in clinical setting for screening of patients at risk of developing financial toxicity and stimulate patient-oncologist cost communication to alleviate the financial burden on cancer survivors.

SFDQ can be incorporated with other patient reported outcome measures in clinical research to study the impact of financial burden on clinical outcomes. However, the provisional SFDQ instrument warrants further larger sample prospective research studies for assessment of validity and other psychometric properties in different primary cancer subsites and treatment modalities from multiple centers to improve the generalizability of this instrument.

## Data Availability Statement

The original contributions presented in the study are included in the article/**Supplementary Material**. Further inquiries can be directed to the corresponding author.

## Ethics Statement

The studies involving human participants were reviewed and approved by the Ethics Committee ICMR-Rajendra Memorial Research Institute of Medical Sciences, Agamkuan, Patna 800007, India. The patients/participants provided their written informed consent to participate in this study.

## Author Contributions

MD conceptualized and designed the study and contributed in data collection, data analysis and drafted the original manuscript. SD was the study supervisor and contributed in data analysis, review and editing the manuscript. RC was study co-supervisor and contributed in patient recruitment, data collection, manuscript writing. KM and VT contributed in manuscript editing and revisions. All the authors contributed in scale item generation. All authors contributed to the article and approved the submitted version.

## Funding

This research did not receive any specific grant from funding agencies in the public, commercial or not for profit sectors.

## Conflict of Interest

The authors declare that the research was conducted in the absence of any commercial or financial relationships that could be construed as a potential conflict of interest.

## Publisher’s Note

All claims expressed in this article are solely those of the authors and do not necessarily represent those of their affiliated organizations, or those of the publisher, the editors and the reviewers. Any product that may be evaluated in this article, or claim that may be made by its manufacturer, is not guaranteed or endorsed by the publisher.
