# Supplementary material for: Development and Validation of Subjective Financial Distress Questionnaire (SFDQ): A Patient Reported Outcome Measure for Assessment of Financial Toxicity Among Radiation Oncology Patients
Source: Front Oncol. 2022 Feb 2;11:819313. doi: 10.3389/fonc.2021.819313 (PMC8847677; doi:10.3389/fonc.2021.819313)
Supplement: Supplementary file 1 [file Table_1.docx]

**Supplementary Table 1:** **Pool of Items associated with subjective financial distress generated through review of literature**

| S. No | Items | Domain | Remarks | Source |
| --- | --- | --- | --- | --- |
| 1 | I know that I have enough money in savings, retirement, or assets to cover the costs of my treatment | FR | Selected (Item 1) | De Souza et al. (2014) |
| 2 | Have you reduced or given up any paid work as a result of your breast cancer | FR | Non-Applicable | Gordon et al. (2007) |
| 3 | Have you or any member of your household had to borrow money from other friends or family members to help pay for your cancer treatment | FR | Selected (Item 5) | Shankaran et al. (2012) |
| 4 | The impact of cancer on your employment | FR | Overlapping | Sharp et al. (2013) |
| 5 | Have you or has anyone in your family had to borrow money or go into debt because of your cancer, its treatment or the lasting effects of that treatment | FR | Overlapping | Yabroff et al. (2016) |
| 6 | I believe being sick will hurt me/has hurt me financially (SWBS) | FR | Overlapping | Meisenberg et al. (2015) |
| 7 | Used all or a portion of savings to pay for cancer care | FR | Selected (Item 3) | Zafar et al. (2013) |
| 8 | Sold possessions or property | FR | Selected (Item 4) | Zafar et al. (2013) |
| 9 | Difficulty living on the total household income | FR | Duplicate | CanCORS |
| 10 | Household ability to make ends meet | FR | Overlapping | Sharp et al. (2016) |
| 11 | Did your health insurance cover you for the costs of everything you expected it would cover in the treatment of your cancer? | FR | Not Applicable | Gordon et al. (2017) |
| 12 | Sought financial assistance from family or friends | FR | Duplicate | Gordon et al. (2017) |
| 13 | Have you reduced or given up any paid work as a result of your cancer treatment costs | FR | Selected (Item 2) | Gordon et al. (2007) |
| 14 | How difficult is it for you to live on your total household income right now | FS | Selected (Item 9) | Pisu et al. (2015) |
| 15 | How difficult is it for you/your family to meet monthly payments on your bills | FS | Overlapping | Abel et al. (2016) |
| 16 | Have you ever been unable to cover your share of medical care visits for cancer and its treatment | FS | Overlapping | Yabroff et al. (2016) |
| 17 | How much of a burden is the cost of overall medical care, including any services needed to cope with cancer | FS | Overlapping | Cagle et al. (2013) |
| 18 | The amount of cash that you have spent on cancer treatment | FS | Overlapping | Chan et al. (2013) |
| 19 | I am currently having difficulty paying for my cancer treatment | FS | Selected (Item 6) | Chan et al. (2013) |
| 20 | My out-of-pocket medical expenses are more than I thought they would be | FS | Selected (Item 8) | De Souza et al. (2014) |
| 21 | In general, how satisfied are you with your family’s present financial situation? | PA | Selected (Item 11) | Abel et al. (2016) |
| 22 | Has your physical condition or medical treatment caused you financial difficulties | PA | overlapping | EORTC QLQ-C30 |
| 23 | Has the financial cost of treating your cancer caused you or your family distress | PA | Selected (Item 12) | Barbaret et al. (2017) |
| 24 | I have more financial distress than physical distress | PA | Overlapping | Barbaret et al. (2017) |
| 25 | How do you feel about your current financial situation? | PA | Overlapping | Bestvina et al. (2014) |
| 26 | My cancer or treatment has reduced my satisfaction with my present financial situation | PA | Overlapping | De Souza et al. (2014) |
| 27 | Worry about wages lost for sick time or attending a medical appointment | PA | Overlapping | Ell et al. (2007) |
| 28 | Would you say your past economic situation is getting better, staying the same or getting worse | PA | Selected (Item 13) | Ell et al. (2007) |
| 29 | Did you experience any financial hardships due to these treatment-related expenses | PA | Duplicate | Goodwin et al. (2013) |
| 30 | Has the financial cost of treating your cancer caused you or your family distress | PA | Duplicate | Gordon et al. (2017) |
| 31 | How often do you worry about being able to meet normal monthly living expenses | PA | Overlapping | Meeker et al. (2016) |
| 32 | I worry about the financial problems I will have in the future as a result of my illness or treatment | PA | Overlapping | De Souza et al. (2014) |
| 33 | How satisfied you are with your present financial situation? (IFD/FWBS) | PA | Duplicate | Meisenberg et al. (2015) |
| 34 | How often do you worry about being able to meet normal monthly living expenses? (IFD/FWBS) | PA | Duplicate | Meisenberg et al. (2015) |
| 35 | How do you feel about your current financial situation? (IFD/FWBS) | PA | Duplicate | Meisenberg et al. (2015) |
| 36 | Did you decide not to have a recommended cancer treatment because it was too expensive | CC | Selected (Item 14) | Barbaret et al. (2017) |
| 37 | I decided to stop the recommended treatment because of the cost | CC | Overlapping | Chan et al. (2013) |
| 38 | When choosing a treatment course for your cancer, did the cost of treatment factor into your decision | CC | Overlapping | Gordon et al. (2017) |
| 39 | Did you decided not to have a recommended test because it was too expensive | CC | Duplicate | Gordon et al. (2017) |
| 40 | Has medical care been delayed for you because of worry about the cost | CC | Overlapping | Kent et al. (2013) |
| 41 | Reduce spending on essential household expenses | CL | Overlapping | Abel et al. (2016) |
| 42 | Sacrificed other things (vacations etc.) (BCFS) | CL | Not Applicable | Meneses et al. (2012) |
| 43 | Changed economic life style (BCFS) | CL | Overlapping | Meneses et al. (2012) |
| 44 | Reduced spending on basics like food and clothing | CL | Selected (Item 15) | Zafar et al. (2013) |
| 45 | Applied for financial assistance programmes provided by government | SS | Overlapping | Chan et al. (2013 |
| 46 | Did you see a financial advisor when you were diagnosed with cancer | SS | Not Applicable | Gorgon et al. (2017) |
| 47 | To what extent have you talked with your primary oncologist about the cost of care | SS | Selected (Item 17) | Meisenberg et al. (2015) |
| **Items associated with subjective financial distress generated through patient interviews** | | | | |
| 1 | Facing difficulties in paying for treatment related travel, food and lodging costs | FS | Selected (Item 7) | Patient Interviews |
| 2 | Stopped working as a result of radiation therapy | FR | Overlapping | Patient Interviews |
| 3 | Currently in debt as a result of cancer treatment expenses | FS | Selected (Item 10) | Patient Interviews |
| 4 | Applied for financial aid schemes (government / non-governmental organizations) | SS | Overlapping | Patient Interviews |
| 5 | The financial assistance and subsidies have eased the financial distress of cancer treatment | SS | Selected (Item 16) | Patient Interviews |

*FR: Financial resources, FS: Financial Spending, PA: Psychosocial Affect, CC: Coping Care, CL: Coping Lifestyle, SS: Support Seeking

**Supplementary Table 2: Preliminary 17-Item instrument for patient administration and data collection**

| **ITEMS** | **SFDQ Items** | **Not at All** | **Some What** | **Very Much** |
| --- | --- | --- | --- | --- |
| Item1 | Do you have sufficient financial resources to cover the costs related to your cancer treatment | 0 | 1 | 2 |
| Item2 | Have you experienced a loss of income/employment/work as a result of your cancer diagnosis or treatment | 0 | 1 | 2 |
| Item3 | Have you used your savings to cover the cost of cancer treatment | 0 | 1 | 2 |
| Item4 | Have you sold any financial assets to cover the cost of cancer treatment | 0 | 1 | 2 |
| Item5 | Have you borrowed money from friends/family to cover the cost of cancer treatment | 0 | 1 | 2 |
| Item6 | Have you faced any difficulties in paying for cancer related treatment costs | 0 | 1 | 2 |
| Item7 | Have you faced any difficulties in paying for treatment related travel, food and lodging expenses | 0 | 1 | 2 |
| Item8 | How much of a financial burden has treatment-related out-of-pocket expenses been on you or your family | 0 | 1 | 2 |
| Item9 | Have you faced any difficulties in paying for daily household expenses due to cancer diagnosis/treatment | 0 | 1 | 2 |
| Item10 | Are you currently in debt as a result of cancer related treatment expenses | 0 | 1 | 2 |
| Item11 | Is your current financial condition satisfactory to you | 0 | 1 | 2 |
| Item12 | Have you or your family experienced financial hardships as a result of your cancer treatment | 0 | 1 | 2 |
| Item13 | Is your financial condition worsening as a result of your cancer diagnosis or treatment | 0 | 1 | 2 |
| Item14 | Have you delayed or avoided treatment, follow-up, or a recommended procedure due to financial concerns | 0 | 1 | 2 |
| Item15 | Have you or your family cut back on essentials like food and clothing as a result of cancer-related expenses. | 0 | 1 | 2 |
| Item16 | Has the financial aid eased the distress due to cancer treatment on you and your family | 0 | 1 | 2 |
| Item17 | Have you discussed about the financial concerns of cancer treatment with your oncologist | 0 | 1 | 2 |
